# Plexiform schwannoma involving the trachea and recurrent laryngeal nerve: a case report

**DOI:** 10.1186/s40792-015-0070-0

**Published:** 2015-08-25

**Authors:** Masashi Nagata, Hiroyuki Ito, Tomohiko Matsuzaki, Hideyuki Furumoto, Tetsuya Isaka, Teppei Nishii, Tomoyuki Yokose, Haruhiko Nakayama

**Affiliations:** Department of Thoracic Surgery, Kanagawa Cancer Center, 2-3-2 Nakao, Asahi-ku, Yokohama, 241-0815 Japan; Department of Pathology, Kanagawa Cancer Center, 2-3-2 Nakao, Asahi-ku, Yokohama, 241-0815 Japan

**Keywords:** Tracheal tumor, Mediastinal tumor, Plexiform schwannoma

## Abstract

Plexiform schwannoma is an infrequent variant of schwannoma characterized grossly and microscopically by multi-nodular growth. Although plexiform schwannoma has such growth patterns, it is a benign tumor as well as a conventional schwannoma. It rarely infiltrates adjacent organs or arises from the organ itself. In this report, we describe a case in which plexiform schwannoma involved the tracheal wall and left recurrent laryngeal nerve to a great extent. As it was expected to be difficult to achieve complete resection even if the longer tracheal resection were performed, we preserved the trachea and resected as much of the tumor as possible. This report is thought to be the first to describe plexiform schwannoma infiltrating or growing from the trachea. Although the treatment decisions we made might be controversial, we believed we could make an accurate diagnosis and adequate treatment decision through surgery.

**Electronic supplementary material:**

The online version of this article (doi:10.1186/s40792-015-0070-0) contains supplementary material, which is available to authorized users.

## Background

Plexiform schwannoma is an infrequent variant of schwannoma characterized grossly and microscopically by multinodular growth [1]. Although plexiform schwannoma has such growth patterns, it is a benign tumor as well as a conventional schwannoma. It rarely infiltrates adjacent organs or arises from the organ itself. In this report, we describe a case in which plexiform schwannoma involved the tracheal wall and left recurrent laryngeal nerve to a great extent. This is thought to be the first case report of plexiform schwannoma infiltrating or growing from the trachea.

## Case presentation

**Fig. 1:**
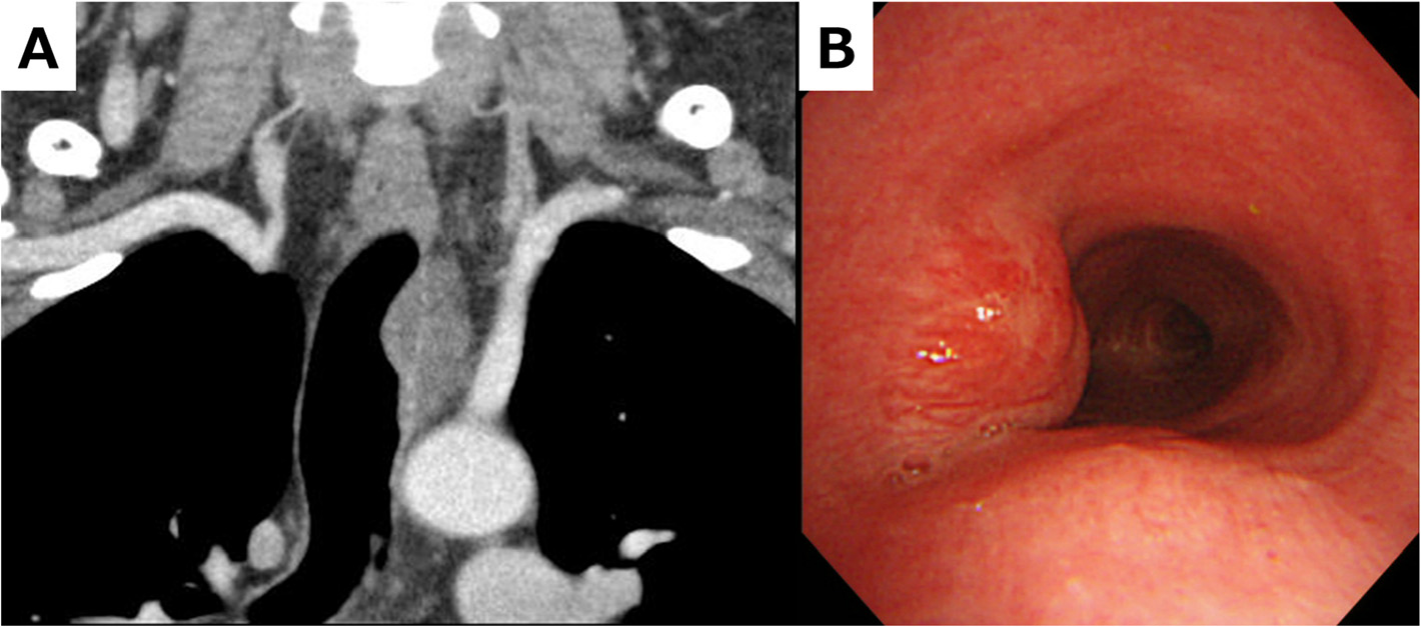
Preoperative findings. **a** Coronal view of chest computed tomography (CT). A tumor involved 3.5 cm of the left tracheal wall, protruding inside and outside the tracheal wall. **b** Bronchoscopy showing a submucosal tumor affecting four tracheal cartilaginous rings

A 39-year-old man with hoarseness and dysphasia was referred to our department for evaluation of left vocal cord palsy and a tracheal tumor that was revealed on computed tomography (CT). He had a previously resected left upper eyelid tumor that was diagnosed as schwannoma. However, he did not have other problems in his medical or family history. Laryngoscopy demonstrated a fixed left vocal cord. CT showed a tumor involving 3.5 cm of the left tracheal wall and protruding inside and outside the tracheal wall (Fig. 1a). Positron emission tomography (PET) presented a maximum standard uptake value of 3.09 on the tumor. There was no accumulation on other parts of the body. Bronchoscopy verified a submucosal tumor affecting four tracheal cartilaginous rings (Fig. 1b). Endobronchial ultrasonographically guided transbronchial needle biopsy and aspiration did not detect malignant tissue and cells.

**Fig. 2:**
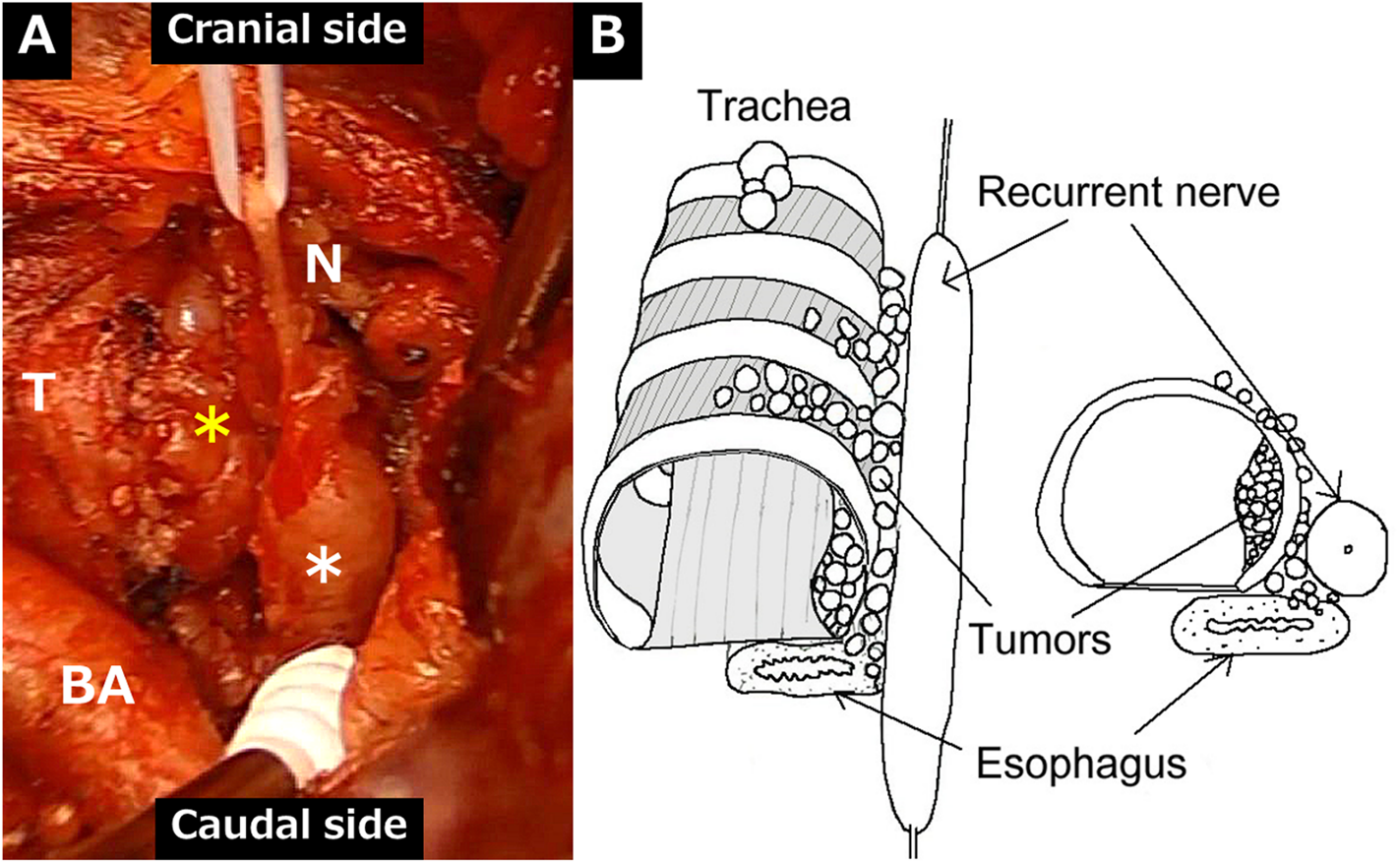
Intraoperative findings. **a** Intraoperative picture. A yellowish soft tumor with multinodularity stretched along the left recurrent nerve (*white asterisk*) and infiltrated the tracheal and esophageal wall (*yellow asterisk*) to a greater extent than estimated. *T* trachea, *N* left recurrence laryngeal nerve, *BA* brachiocephalic artery. **b** Schema of the tumor

**Fig. 3:**
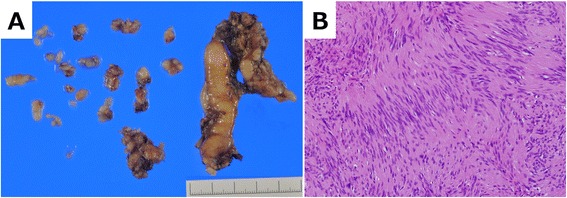
Pathological findings. **a** Macroscopic specimen of the resected tumors. **b** Hematoxylin and eosin stain (×100). There were spindle cells shown in a palisaded fashion. It was a typical finding of Antoni A schwannoma with no malignancy

Although a preoperative diagnosis could not be made, the tumor was suspected to be malignant. If the tumor were to become larger, tracheal resection for complete removal would become impossible due to associated risks. Therefore, we performed median sternotomy to explore and diagnose the tumor with a subsequent planned tracheal resection if indicated and possible. Informed consent from the patient and his family was obtained. Surgical exploration revealed a yellowish, soft, multinodular tumor stretching along the left recurrent nerve and infiltrating the tracheal and esophageal walls (Fig. 2a, b). The left recurrent nerve involved with the tumor was transected, and an incisional biopsy was performed. The intraoperative pathological diagnosis was schwannoma without malignancy. This tumor was much more widespread than expected. The tumor invaded outer tracheal wall over four tracheal rings. However, submucosal infiltration seemed to be developed more. We confirmed small nodules on the cranial side of tracheal wall apart from the main tumor, which were also diagnosed as schwannoma (Fig. 2b). The invasion to esophageal wall could not be anticipated from CT findings. As it was impossible to achieve complete resection, we determined to preserve the trachea and resect the tumor nodules to the greatest extent possible (Fig. 3a). Additional movie files can show this operation in more detail [see Additional files 1 and 2].

The pathological features were identical to those of typical schwannomas, including being composed solely of Schwann cells frequently disposed in a compact, palisaded fashion (Antoni A) (Fig. 3b) and occasionally in a loose texture (Antoni B). This tumor did not have cellular atypia, hyperchromasia either. Cellularity was not high. There was no remarkable pleomorphism. We could confirm few mitotic activities. Immunohistological staining for S-100 protein showed diffuse positivity, while that for neurofilament showed weak positivity only in peripheral areas, which is atypical for a neurofibroma. The patient’s postoperative course was uneventful. A lump on the trachea remained, but no further growth was observed in an 8-month postoperative follow-up period.

### Comment

Taking the findings for this tumor into consideration, we can postulate two hypotheses about how this plexiform schwannoma progressed. First, it is possible that there were two individual tumors—one arising from the left recurrent laryngeal nerve and the other from the trachea itself. Second, the plexiform schwannoma may have originated from the recurrent nerve and infiltrated the tracheal wall. Whichever postulation is true, such an occurrence is very rare.

Plexiform schwannomas have been reported to represent 4.3 % of all schwannomas, and they are often seen in the head and neck region [1]. Only one case of tracheal plexiform schwannoma was described in a case series reporting uncommon primary tracheal tumors [2], and it was not presented in detail. In a literature search using PubMed, we could not find any description of a plexiform schwannoma originating from the recurrent laryngeal nerve or of a plexiform schwannoma infiltrating the tracheal wall. In one report, the association of plexiform schwannoma with neurofibromatosis type 2 and with schwannomatosis was 5 % each [1]. Our patient did not have a history of bilateral vestibular schwannomas or of tumors arising from the central nervous system, such as meningiomas. He also did not have a family history suggesting neurofibromatosis. Schwannomatosis has recently been recognized as the third major form of neurofibromatosis that causes multiple schwannomas without a diagnosis of neurofibromatosis type 2 [3]. Some researchers have reported genetic alterations in this disorder [4]. However, the diagnostic criteria have not yet been established. Our patient had a history of left eyelid schwannoma, and he may be diagnosed with schwannomatosis in the future.

If repeated needle biopsies or resection of the intraluminal bulge under rigid bronchoscopy had proven schwannoma before surgery, we might avoid trying the risky tracheal resection and observe the tumor unless tumor growing or tracheal stenosis occurred. However, at that time, it was not expected that the tumor was a benign tumor, such as schwannoma, and a repeat biopsy was not done. We therefore determined that we could make an accurate diagnosis and adequate treatment decision through surgery. After the intraoperative diagnosis, we determined to preserve the trachea and resect as much of the tumor as possible. Wright and coworkers argued that tracheal resection by more than 4 cm was a significant risk factor for anastomotic complications, although there were some measures to loosen the tension [5]. In the present case, even if we had attempted the longer tracheal resection, there would be no guarantee that we would not leave a tumor at the tracheal stump. Residual plexiform schwannoma can recur. Recurrent plexiform schwannomas were reported even with such a high-risk surgery [6, 7]. Moreover, as the tumor had infiltrated a part of the esophageal wall, we considered that it would be impossible to achieve curative resection. Plexiform schwannoma can become enlarged, but it occurs slowly. Debulking is supposed to extend significantly the progression-free period. Periodic follow-up must continue in order to check for regrowth. If this tumor grows up intraluminally, we will resect it under rigid bronchoscopy.

Pathological diagnosis of this tumor was typical schwannoma without malignant findings. Some of plexiform schwannoma are difficult to be differentiated from malignant peripheral nerve sheath tumor [8]. With detailed pathological findings about cellularity and mitotic activity, this tumor could clearly be distinguished from malignancy. Therefore, the prognosis of this case is expected to be good though local recurrence may happen. A few malignant transformations were reported in familial schwannomatosis [9]. It is unknown whether the plexiform schwannoma in this patient may transform to the malignancy. We should suspect of malignant transformation when we see the rapid enlargement.

## Conclusions

We experienced an extremely rare case of plexiform schwannoma involving the tracheal wall and left recurrent nerve to a great extent. Appropriate treatment was difficult to determine. Periodic follow-up must continue in order to check for regrowth.

## Consent

The informed consent was obtained from the patient for the publication of this report and any accompanying images. A copy of the written consent is available for review by the Editor-in-Chief of this journal.

## Additional files

Additional file 1:
**This movie file is the first half of the digest showing this surgery.** (MP4 15357 kb) Additional file 2:
**This movie file is the latter half.** (MP4 13857 kb)
